# Homotypic Transposable Element Pairing May Drive Coherent Chromatin Folding

**DOI:** 10.3390/genes17010043

**Published:** 2025-12-31

**Authors:** Max Myakishev-Rempel

**Affiliations:** 1 DNA Resonance Research Foundation, San Diego, CA 92111, USA; mrempel@dnaresonance.org; 2 DNA Vibe LLC, Centennial, CO 80112, USA

**Keywords:** transposable elements, chromatin, chromatin conformation, Micro-C, Hi-C, MIR, L2, Alu

## Abstract

**Background/Objectives:** Transposable elements comprise over 50% of the human genome, yet their role in chromatin organization is insufficiently studied. This study was motivated by the hypothesis that transposable elements drive chromatin contacts through homotypic coupling—that is, pairs of identical TEs physically pull genomic regions together. **Methods:** Analyzing public Micro-C and Hi-C datasets, I compared focal contact areas that stand out from low backgrounds against contact-depleted regions at kilobase resolution. **Results:** I discovered that transposable elements show enrichment at these focal contact points and even stronger depletion in contact-poor regions. Ancient mammalian transposable element families (MIR, L2) preferentially form homotypic pairs at chromatin contacts, while young primate-specific families (Alu, SVA) actively avoid homotypic pairing. The depletion of homotypic pairs in contact-poor regions exceeded their enrichment at focal contacts, suggesting that homotypically coupled transposable elements may be sequestered in protein-protected compartments. Unexpectedly, sequence-unrelated families like MIR and L2 showed similarly strong pairing behavior, indicating a mechanism independent of DNA sequence similarity. While my data demonstrates clear homotypic specificity patterns reproducible across cell types and individuals, it cannot distinguish whether transposable elements actively drive chromatin contacts or are passive markers of chromatin states. **Conclusions:** Study findings reveal previously uncharacterized patterns of transposable element organization at chromatin contact and suggest that homotypic pairing may provide anchoring points for coherent chromatin folding.

## 1. Introduction

Transposable elements comprise over 50% of the human genome and contribute to chromatin organization [1,2]. Recent work demonstrated that transposable elements form homotypic clusters in nuclear space—meaning that L1 elements preferentially associate with other L1s and Alu elements associate with other Alus [3]. This homotypic clustering creates distinct three-dimensional compartments visible by microscopy. In my previous work, I proposed that transposable elements may function as structural anchors that facilitate and control chromatin folding [4,5,6,7,8,9,10,11].

This study was motivated by my hypothesis that transposable elements drive chromatin contacts through homotypic coupling—identical TEs physically glue genomic regions together. To address this, I analyzed public chromatin conformation capture data to map homotypic coupling patterns at chromatin contacts, comparing focal contacts (hotspots) against contact-depleted regions (coldfields) at kilobase resolution.

## 2. Methods

### 2.1. Chromatin Conformation Datasets

I analyzed chromatin conformation capture data from eight human datasets, referenced by their 4D Nucleome Data Portal (https://data.4dnucleome.org, accessed on 1 July 2025). Provided are nicknames used in this paper and accession numbers. Pairs.gz files were downloaded from the 4D Nucleome Data Portal [12,13].

Micro-C datasets [14]

- ZITI (4DNFI1O6IL1Q): H1-hESC stem cells, Caucasian male, Micro-C (MNase);
- WALNUT (4DNFI3ULPBI5): WTC-11 iPSC cells, Japanese male, Micro-C (MNase);
- ALMOND (4DNFIEVQW3GY): WTC-11 iPSC cells, Japanese male, Micro-C (MNase);
- PASTRY (4DNFICOEXGPJ): HFFc6 immortalized fibroblasts, Micro-C (MNase);

Hi-C dataset [15]: BREAD (4DNFIYECESRC): HFFc6 immortalized fibroblasts, in situ Hi-C (MboI).

### 2.2. Chromatin Contact Data Processing, Selecting Hotspots and Coldfields

I analyzed Micro-C data from H1-ESC human embryonic stem cells (dataset 4DNFI1O6IL1Q). I processed paired-end reads to identify chromatin contacts between 8 and 40 kb distances. This range captures local loops while avoiding immediate neighbors.

### 2.3. Hotspot Identification

I divided the genome into 600 bp bins. For each bin pair with contacts, I extracted a 5 × 5 grid centered on that position in the 2D contact matrix. I excluded the 4 corners, leaving 21 bins. A position was qualified as a hotspot when the center bin had ≥5 contacts, exceeded the second-highest value by ≥20%, and the median was <40% of the center. These criteria identified focal enrichments rather than diffuse regions. These thresholds were chosen to ensure statistical reliability while capturing focal contacts; the strong effect sizes observed (hot/cold ratios of 1.5–2.0) indicate robustness to reasonable parameter variation.

### 2.4. Coldfield Identification

For each hotspot, I searched diagonally for its coldfield partner. Starting adjacent to the hotspot (0-bin offset), I positioned 5 bins diagonally and checked 20 positions in each direction. This covered ±57 kb from the hotspot. At each position, I evaluated a 5 × 5 grid using the TMC metric: (max^1.3^) × median. The position with the lowest TMC became the paired coldfield.

### 2.5. Data Sampling

Due to file size, I sampled systematically. I skipped the first 1 M read pairs. Then I processed 800 K consecutive pairs, skipped 2.5 M pairs, and repeated. Each hotspot is paired with exactly one coldfield. Output contained paired coordinates with contact counts, enrichment ratios, and TMC scores. As a control to exclude artifacts from my algorithm or analysis pipeline, I repeated the second phase analysis using artificially incorrect 580 bp bin coordinates instead of 600 bp bins. This parameter effectively reshuffled the coordinates and, as a result, completely eliminated the focal signal position zero, confirming that the signal is not a computational artifact. This sampling strategy analyzed 50 M read pairs (approximately 25% of the dataset), yielding 700 K contact pairs and identifying 64 K hotspots with matched coldfields. Results were consistent across different sampling runs.

### 2.6. Reproducibility of TE Homotypic Pairing at Contacts

For the reproducibility analysis (Section 3.3), I analyzed homotypic pairing patterns of the 30 most abundant TE families from DFAM annotations [16]. Homotypic ratios were calculated as homotypic counts in hotspots divided by homotypic counts in coldfields within the central 2 kb window around contact points. Pearson correlations were computed between datasets. Hierarchical clustering used average linkage with Euclidean distance.

### 2.7. TE Pairing Specificity Measurement

I mapped transposable elements (TEs) around each hotspot and coldfield using the DFAM database indexed for hg38. I divided the genome into 10 kb bins for TE indexing. For each contact point, I extracted TEs within ±11 kb.

I used 21 sliding windows of 2 kb width with 50% overlap, spanning from −11 kb to +11 kb around each contact point. The center window covered −1 kb to +1 kb. For each window, I collected all TEs whose centers fell within that range.

At each hotspot, I compared TEs at the two contact anchors (the paired genomic positions forming the contact). When the same TE family appeared at both anchors, I counted it as a homotypic pairing. When different families appeared, I counted heterotypic pairing. I performed the same analysis for coldfields.

I calculated three metrics for each TE family. First, the hot/cold ratio: homotypic counts in hotspots divided by homotypic counts in coldfields. Second, the heterotypic ratio: heterotypic counts in hotspots divided by heterotypic counts in coldfields. Third, the specificity index: hot/cold ratio divided by heterotypic ratio. This specificity index measures preference for same-family pairing versus different-family pairing. This ratio inherently controls for local TE density, as both numerator and denominator scale with element abundance. Additionally, comparing hotspots against matched coldfields controls for regional density variation.

I analyzed the top 30 TE families by abundance. For visualization, I plotted the top 6 families based on total counts across all windows. The analysis included ALU, L1, L2, MIR, and ERVL families, among others. TE strand orientation showed no significant effect on homotypic pairing (head–head to head–tail ratio = 0.94). Element length was not tested separately.

## 3. Results

### 3.1. Study Design: Identifying Focal Contacts Versus Diffuse Regions

**Figure 1:**
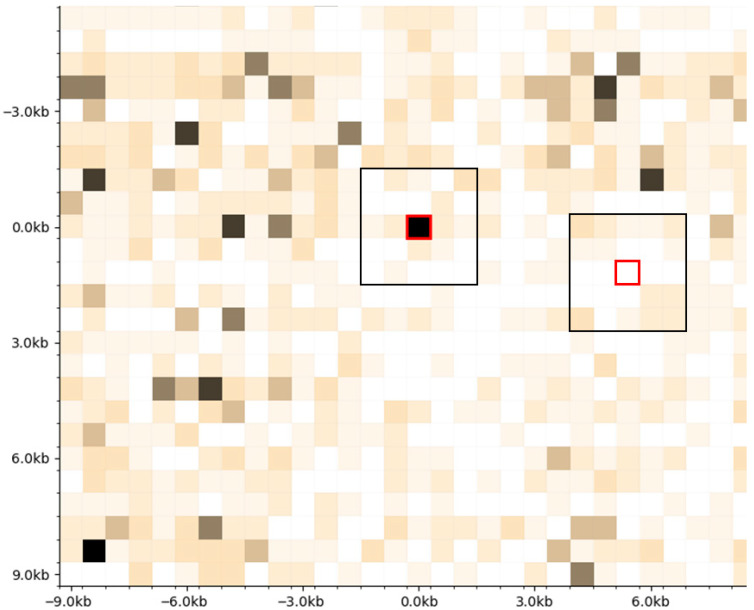
Selection of hotspots and coldfields. Hotspots are selected to be singular high-count 600 × 600 bp bins standing out from low background, and coldfields are selected to be low-count bins on a low-count background. Red box: hotspot; black box: matched coldfield.

Chromatin contact heatmaps contain extensive fields of high contact frequency where contact positions likely shift between cells. This variability makes it difficult to map sequence specificity using all contacts within these dense regions. I therefore focused on hotspots—singular high-count bins that stand out from low-count backgrounds (Figure 1). For each hotspot, I identified a matched coldfield control by searching diagonally for regions with low overall counts in 3 × 3 kilobase windows and low central counts. I called these regions coldfields rather than coldspots because they represent extended areas of contact depletion.

### 3.2. Transposable Element Distribution at Chromatin Contacts

I analyzed Micro-C data at 300 base pair resolutions to investigate how transposable elements distribute around chromatin contacts. I examined transposable element density in 600 base pair bins, first measuring the raw density of individual elements before testing for homotypic interactions.

**Figure 2:**
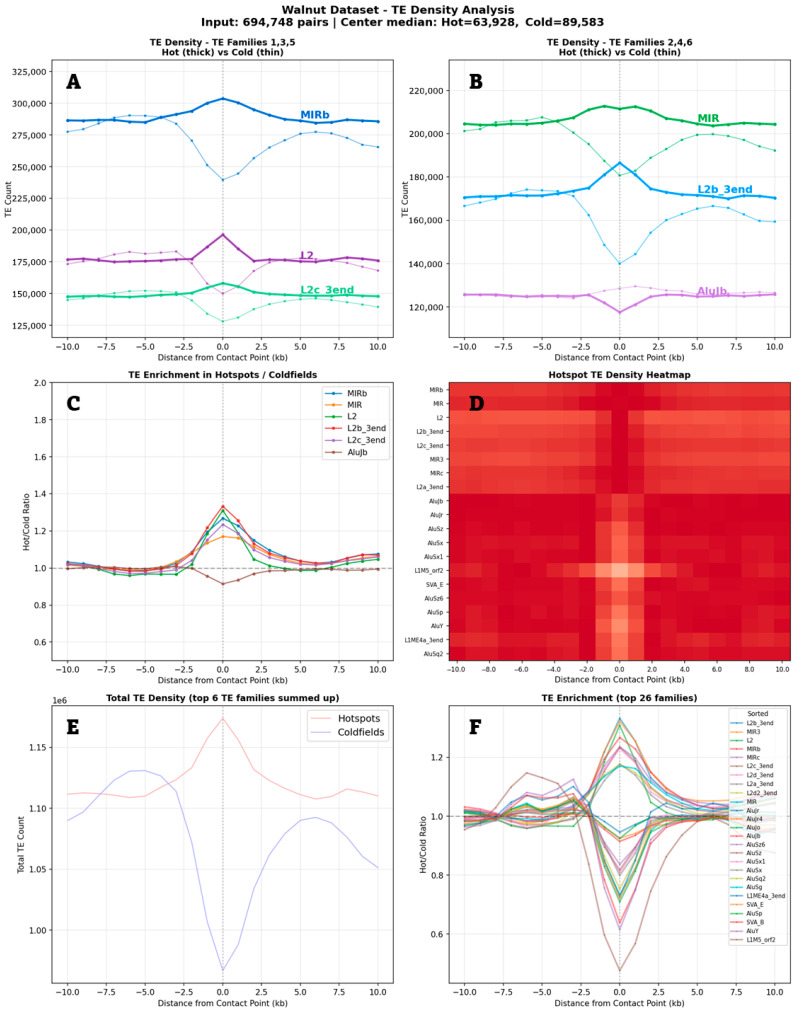
TE density distribution near chromatin contact points. H1-ESC Micro-C dataset analyzed with 2 kb sliding windows. Distance 0 = contact point. (**A**,**B**) Raw TE counts for top families at hotspots (thick lines) vs. coldfields (thin lines). (**C**) Hot/cold enrichment ratios. (**D**) Hotspot density heatmap. (**E**) Total TE density summed across the top 6 families. (**F**) Enrichment profiles for the top 26 families.

Individual transposable elements showed distinct distribution patterns around contact points (Figure 2). Most transposable element families showed modest enrichment at hotspot centers, with MIRb displaying the strongest focal peak. In coldfields, I observed sharp central depletion for all transposable element families. L2 and MIR elements showed particularly pronounced depletion. The depletion in coldfields exceeded the enrichment in hotspots. This asymmetry was unexpected—the coldfield signal proved stronger than the hotspot signal.

### 3.3. Homotypic Pairing at Chromatin Contacts

I next tested whether regions brought together at chromatin contacts share sequence homology through their transposable elements. I scored homotypic pairing when the same transposable element family appeared on both genomic locations (sides) that come in contact. I also scored heterotypic pairing when different families were present.

**Figure 3:**
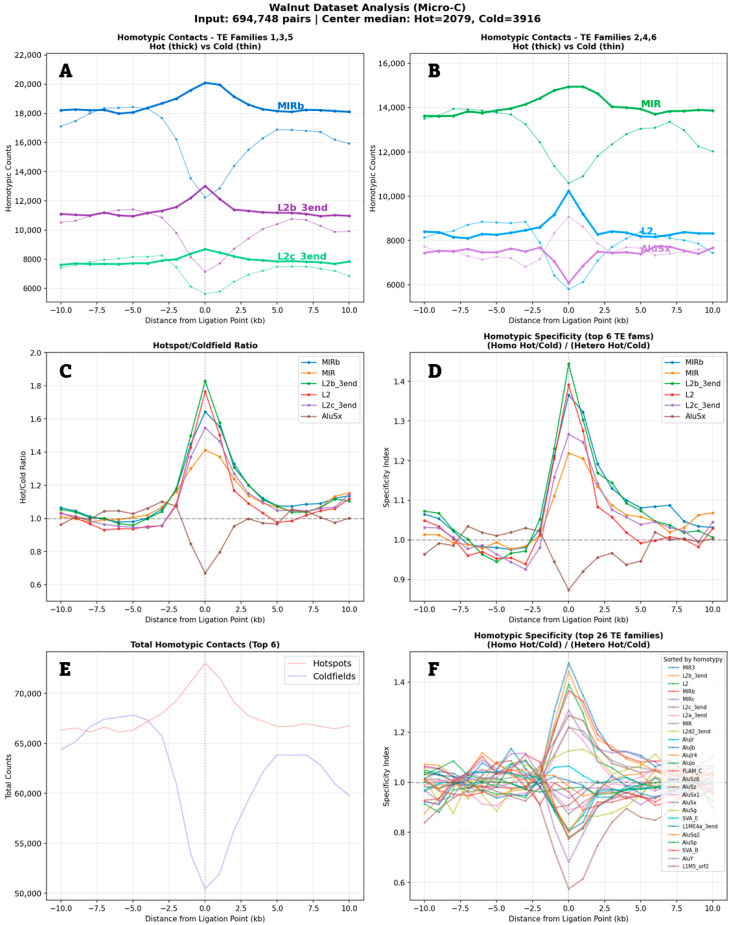
Homotypic specificity of TEs at chromatin interaction hotspots versus coldfields. (**A**,**B**) Raw homotypic counts for the top 6 TE families. Thick lines: hotspots; thin lines: coldfields. (**C**) Hot/cold count ratios. (**D**) Specificity index (homotypic/heterotypic ratio) for the top 6 families. (**E**) Summed counts of the top 6 families, hot vs. cold. (**F**) Same as panel (**D**), but for the top 26 families.

The homotypic pairing analysis revealed an unexpected pattern (Figure 3). The curves look nearly identical to the raw density curves. This means that when a transposable element appears at a contact point, another transposable element of the same family appears on the other side of that contact. The peak at position zero—the exact contact point—shows this is where identical transposable elements meet. In hotspots, homotypic counts go up. In coldfields, they drop strongly. The drop is deeper than the peak is high.

This pattern holds for ancient transposable element families like MIR and L2. These families preferentially pair with themselves at hotspots. Young Alu families do the opposite—they avoid pairing with themselves. The sharp peak exactly at the contact point tells us this is not some broad regional effect. Something specific happens at the contact point. The resolution of the micro-C method is around 300 bp, and therefore, to obtain sufficient data, I chose to work with 600 bp bins. That allowed me to map the peaks with a half-width of around 3 kb.

### 3.4. Reproducibility of Homotypic Pairing Patterns

**Figure 4:**
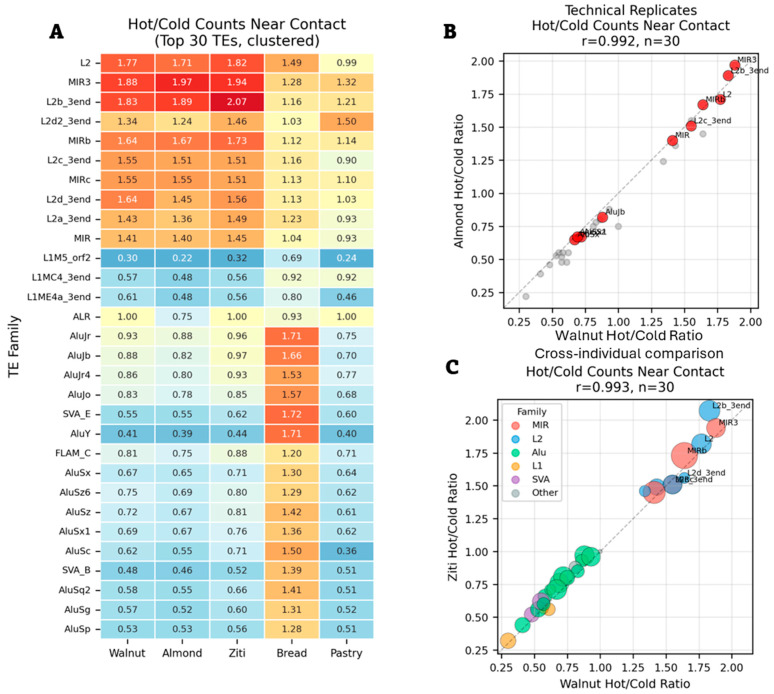
Reproducibility of TE homotypic pairing at contacts. (**A**) Hot/cold ratios for top 30 TE families across five datasets, hierarchically clustered. Values show homotypic enrichment (red) or depletion (blue) at hotspots relative to coldfields. (**B**) Technical replicates (Walnut vs. Almond) show near-perfect correlation (r = 0.992). (**C**) Cross-individual comparison (Walnut vs. Ziti) maintains high correlation (r = 0.993), with families color-coded by superfamily.

I tested whether homotypic pairing patterns were consistent across datasets. I analyzed homotypic ratios for the 30 most abundant transposable element families across multiple cell types and individuals (Figure 4).

Technical replicates showed near-perfect correlation. The same patterns appeared when comparing different individuals. This reproducibility demonstrates that transposable element pairing patterns are not random but reflect underlying chromatin organization principles.

L2 and MIR families consistently showed strong homotypic pairing across all datasets. Alu and SVA families consistently avoided homotypic pairing. This binary distribution—ancient families pair, young families avoid pairing—held across all cell types tested.

I compared Hi-C and Micro-C data from the same cell line. The methods showed opposite patterns. Where Micro-C showed homotypic enrichment, Hi-C showed depletion. This negative correlation indicates that these two methods capture different aspects of chromatin organization. This reflects methodological differences: MNase (Micro-C) produces ~150 bp fragments while restriction enzymes (Hi-C) produce fragments of several hundred base pairs. These radically different fragmentation sizes capture different populations of chromatin contacts.

### 3.5. Pairwise Specificity and Evolutionary Age Correlation

**Figure 5:**
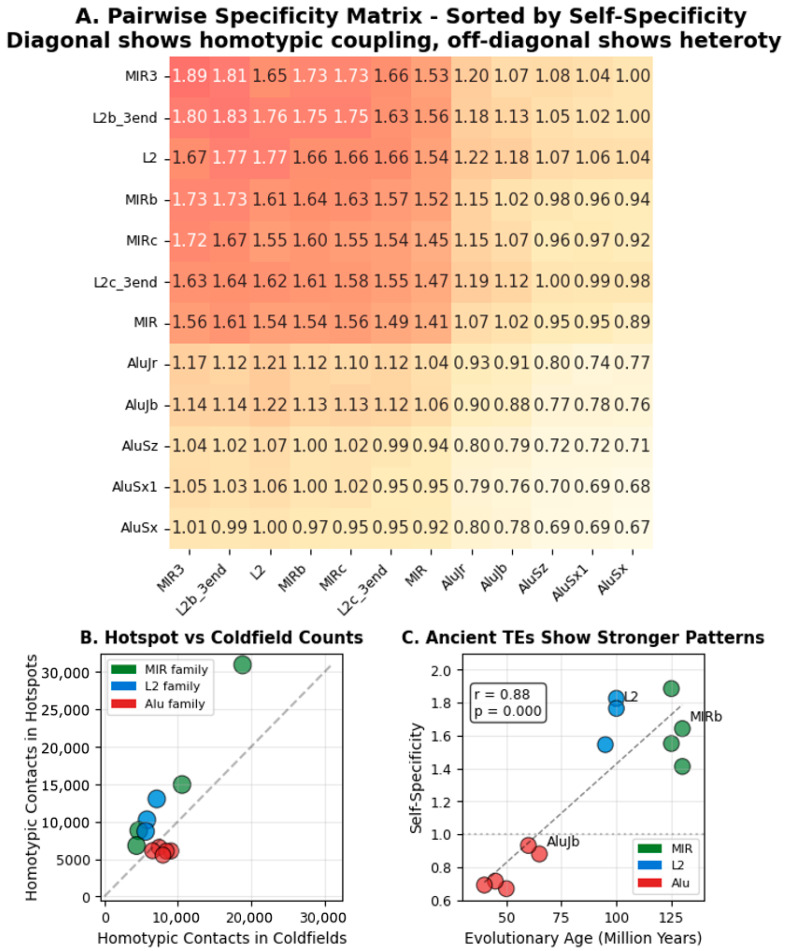
Homotypic specificity at chromatin contacts. (**A**) Pairwise specificity matrix showing hot/cold homotypical count ratios for 12 TE families, sorted by self-specificity (diagonal values). Values > 1.0 indicate enrichment in chromatin interaction hotspots relative to coldfields. Values < 1.0 indicate depletion. (**B**). Homotypic contact counts at ligation junctions comparing hotspots versus coldfields. Points above the diagonal indicate homotypic specificity. (**C**) Correlation between TE evolutionary age and homotypic specificity. Ancient families (MIR, ~130 Myr) show higher homotypic specificity than younger families (Alu, ~50 Myr). Walnut Micro-C dataset.

I examined whether transposable element families show pairwise specificity for pairing with themselves versus other families (Figure 5). The pairwise specificity matrix reveals distinct patterns based on evolutionary age.

Ancient MIR families showed the strongest self-preference—they pair with themselves more than with any other family. Higher numbers presented on the heatmap diagonally indicate this self-specificity. L2 families also showed strong homotypic preference. These families diverged early in mammalian evolution, over 130 million years ago [17].

Young Alu elements showed the opposite pattern. They actively avoid pairing with themselves near contacts. The matrix shows negative values for Alu self-pairing. Alus emerged later, around 50 million years ago in primate evolution [18].

The off-diagonal values in the matrix reveal something unexpected. Sequence-unrelated families like MIR and L2 show high cross-coupling. Despite having completely different DNA sequences, when MIR appears on one side of a contact and L2 on the other, these heterotypic pairs show the same pattern as homotypic pairs—increased counts at hotspots, decreased counts at coldfields. MIR-L2 pairs behave just like MIR-MIR or L2-L2 pairs. This convergent behavior suggests they share a common mechanism that does not depend on DNA sequence similarity.

The correlation between evolutionary age and homotypic specificity was strong. The older the transposable element family, the more it prefers to pair with itself. This age gradient spans from ancient mammalian elements to recent primate-specific insertions. The gradient suggests that transposable elements have been progressively co-opted into chromatin organization throughout evolution.

## 4. Discussion

### 4.1. Interpreting the Sharp Focal Signal

I observed sharp homotypic transposable element depletion at coldfield contact points and enrichment at hotspot contact points. The signal centers at zero and decays within 3 kb. Such 3 kb-scale peaks and antipeaks of TEs and their homotypic coupling have not been previously examined in chromatin contact studies, which typically analyze broader compartments at megabase scales.

### 4.2. Two Interpretations of the Depletion Signal

The stronger depletion in coldfields compared to enrichment in hotspots suggests two possible mechanisms. First, certain families of transposable elements might simply mark free DNA compartments. Second, homotypically coupled transposable elements might actively sequester DNA into protein-protected compartments that resist nuclease digestion. If homotypic pairs were present at coldfield positions, they would protect the DNA from cleavage, preventing contact detection. Sequence-specific bridging proteins that recognize transposable element motifs could mediate the coupling.

These interpretations are not mutually exclusive. Transposable elements could both mark and drive chromatin organization. The distinction matters for understanding causality but does not change the observation that homotypic pairing correlates strongly with contact detection.

### 4.3. The MIR-L2 Cross-Specificity Problem

The pairwise specificity matrix shows that MIR and L2 families behave similarly despite sequence divergence. MIR-L2 heterotypic pairs show nearly the same hot/cold ratio as MIR-MIR or L2-L2 homotypic pairs. This cross-specificity suggests that sequence identity alone does not determine pairing behavior.

Several mechanisms could explain this cross-family coupling. Both families might recruit the same sequence-specific bridging proteins that dimerize to couple distant sites, similar to how CTCF or HP1 proteins bring together chromatin regions. Such proteins could actively pull the coupled transposable elements into compacted, protein-protected domains. This would explain both the homotypic coupling and the protection from nuclease digestion. Alternatively, they might share similar methylation patterns that create comparable chromatin environments or occupy the same specialized nuclear compartments. These possibilities remain to be tested.

### 4.4. The Cause–Consequence Question

While my data cannot definitively prove whether transposable elements actively drive contacts or passively mark chromatin states, several observations are consistent with an active role. The sharp focal signal argues against simple compartmentalization. The consistent patterns across cell types suggest functional constraints. One possibility is that homotypic TE pairing may provide sequence-specific anchoring points for hierarchical chromatin folding—enabling orderly stepwise packaging rather than chaotic contacts. Further research is needed to confirm this mechanism.

### 4.5. Distinguishing Markers from Drivers

Further conservation analysis could help distinguish passive markers from active drivers. If transposable elements merely mark pre-existing chromatin states, their positions should evolve neutrally. If they actively drive chromatin organization through recruited bridging proteins or other mechanisms, their positions should show evolutionary conservation. Active drivers would face stronger selection pressure to maintain their genomic positions.

## Data Availability

The custom Python 3 scripts developed in this study are openly available on GitHub https://github.com/maxrempel/lp2, accessed 30 December 2025.
